# Inhibition of AGS human gastric cancer cell invasion and proliferation by *Capsosiphon fulvescens* glycoprotein

**DOI:** 10.3892/mmr.2013.1492

**Published:** 2013-05-27

**Authors:** YOUNG-MIN KIM, IN-HYE KIM, TAEK-JEONG NAM

**Affiliations:** Institute of Fisheries Sciences, Pukyong National University, Ilgwang-ro, Ilgwang-myeon, Gijang-gun, Busan 619-911, Republic of Korea

**Keywords:** *Capsosiphon fulvescens*, tight junction protein, cell invasion

## Abstract

Seaweeds are increasingly being used as foodstuffs and therapeutics. *Capsosiphon fulvescens* (*C. fulvescens*) is a green sea alga which has demonstrated anti-tumor activity in various cancer cell lines. In cancer cells, homeostasis is not maintained, enabling mutations to develop and growth to continue unchecked. Overexpression of matrix metalloproteinase (MMP) and tight junction (TJ) proteins is important for cancer cell proliferation, invasion and metastasis. In addition, these proteins are closely associated with cell membrane permeability. In the current study, *C. fulvescens* glycoprotein (Cf-GP) was found to inhibit TJ proteins and invasion of AGS human gastric cancer cells. Cf-GP-mediated inhibition of cell proliferation and invasion was confirmed, as well as changes in TJ protein levels. In addition, MMP-2 and −9 activities were inhibited, as indicated by increased transepithelial electrical resistance. Inhibition of MMP protein expression was also found to correlate with tissue inhibitor of metalloproteinase 1 and Cf-GP treatment, as revealed by western blot analysis and RT-PCR. In conclusion, these results indicate that Cf-GP inhibits cancer cell invasion and therefore demonstrates a potential therapeutic strategy to decrease cancer metastasis.

## Introduction

Cells become cancerous as a result of mutation of normal genes, resulting in continuous growth and survival. Human gastric cancer is linked to irregular eating habits and intake of fast food (1). In recent years, studies on cancer cell apoptosis and inhibition of cancer cell invasion has been conducted (2). In particular, various seaweeds have been reported to inhibit cancer cell growth (3–5). The majority of studies on seaweeds are conducted in Japan and Korea (6,7). Anti-tumor activity in HT-29 colon cancer cells using *Laminaria japonica*(8) and the recovery of damaged liver cells using *Hizikia fusiformis* (*H. fusiformis*) glycoprotein have been previously reported (9). In addition, inhibition of the growth of AGS human gastric cancer cells by a *H. fusiformis* extract has been noted (10).

*Capsosiphon fulvescens* (*C. fulvescens*) is a green alga that grows in clean areas off the Korean coast. *C. fulvescens* has long been a traditional Korean food and exhibits anti-cancer effects in addition to improving hangover symptoms, immune activity and anticoagulant activity. Anti-cancer effects of *C. fulvescens* components have been reported (11,12). Prior to the present study, *C. fulvescens* glycoprotein treatment was reported to induce apoptosis of human gastric cancer AGS cells via Fas signaling (13). Increased β-catenin levels increase cell adhesion, leading to invasion and metastasis. These processes are accelerated in cancer cell metastasis and invasion mediated by adhesion between adjacent cancer cells; this is facilitated by increased β-catenin levels. Although cancer cell apoptosis is induced by Fas, β-catenin levels were decreased. Therefore, apoptosis was examined via Fas signaling; however, as invasion was not inhibited, further experiments were conducted.

The inhibitory effect of *C. fulvescens* on invasion by human gastric cancer AGS cells was also evaluated. Tight junction (TJ) and matrix metalloproteinase (MMP) proteins are overexpressed in a number of cancer cells and are associated with their invasive properties (14). Although MMP and TJ protein levels are increased, the transepithelial electrical resistance (TEER) value is lower. TEER facilitates material transfer between cells due to disrupted cell membrane permeability (15,16). In addition, cancer cell adhesion, metastasis, proliferation and invasion increased. Therefore, studies of apoptosis signaling in cancer cells, as well as invasion and metastasis, have been performed recently. Therefore, in the present study, TJ proteins, claudin, zo-1 and occludin, and the cell adhesion-related molecules, β-catenin and E-cadherin, were investigated. *C. fulvescens* glycoprotein (Cf-GP) controls protein expression, which is associated with cancer cell invasion. Results indicate that Cf-GP treatment inhibits invasion and development of AGS human gastric cancer cells.

## Materials and methods

### Preparation of Cf-GP

Cf-GP was purchased in 2006 in Korea. *C. fulvescens* powder (40 g) was diluted in 1 liter water and stirred for 3 h at 80°C using a heating mantle, followed by centrifugation at 1,500 × g for 15 min at 4°C.

Three volumes of 95% ethanol were added and precipitate was removed by vacuum filtration. To the supernatants, 80% ammonium sulfate was added, followed by stirring for 24 h. Next, salt was removed by membrane dialysis (Por Membrane MW 3,500 Da, Spectrum Laboratories Inc., Rancho Dominguez, CA, USA) for 1 day at 4°C. The concentrated solution was aliquoted into 1.5 ml tubes and stored at −70°C until use. These samples are hereafter termed Cf-GP.

### Cell culture

Human gastric cancer AGS cells (American Type Culture Collection, Manassas, VA, USA) were maintained at 37°C in a 5% CO_2_ humidified atmosphere. Cells were cultured in RPMI-1640 medium with 10% fetal bovine serum (FBS; Hyclone, Logan, UT, USA), 100 U/l penicillin and 100 mg/l streptomycin. Cells were cultured to 60–80% confluence in 100-mm diameter dishes. The medium was replaced every day.

### Cell proliferation assays

AGS cell proliferation was measured using a CellTiter 96^®^ aqueous non-radioactivity cell proliferation assay (Promega Corporation, Madison, WI, USA). The assay is based on the cleavage of 3-(4,5-dimethylthiazol-2-yl)-5-(3-carboxymethoxy-phenyl)-2-(4-sulfonyl)-2H-tetrazolium (MTS) into a formazan product that is soluble in tissue culture media. Cells were seeded onto 96-well plates at 2×10^4^ cells/well in 100 μl medium. Cells were maintained for 24 h and the medium was then replaced with serum-free medium (SFM). Following 24 h, the medium was replaced with SFM containing Cf-GP (0, 5, 10 or 20 μg/ml) for 24 h. Cells were then incubated with MTS solution for 30 min at 37°C. Cell proliferation was measured by means of absorbance at 490 nm using the Benchmark enzyme-linked immunosorbent assay (ELISA) plate reader (Bio-Rad, Hercules, CA, USA).

### Cell invasion assays

Cell invasion was measured using an 8.0-μm pore size insert in Transwell^®^ plates (Corning Costar Inc., Corning, NY, USA). AGS cells were seeded into the upper chamber (Matrigel coated; Corning Costar Inc.) and maintained for 24 h. The medium was then replaced with SFM containing Cf-GP (0, 5, 10 or 20 μg/ml) for 24 h. The lower chamber was maintained with 10% FBS. Following 24 h, the cut on the bottom of the filter (Matrigel coated) was stained with hematoxylin. Stained cells were calculated by extrapolation from the number counted.

### TEER assay

Using a voltohmmeter (EVOM Epithelial Tissue Voltohmmeter; World Precision Instruments, Sarasota, FL, USA), TEER was measured. AGS cells were seeded into the upper chamber (Matrigel coated) of the transwell plates and maintained for 24 h. Next, SFM was replaced with medium containing Cf-GP (0, 5, 10 or 20 μg/ml) for 24 h. The lower chamber was maintained with 10% FBS. Following 24 h, the upper chamber was separated and TEER values were determined using the voltohmmeter.

TEER was calculated using the following formula: TEER [(resistance (Ω cm^2^)] = (Ω - background Ω) × membrane area (cm^2^); background resistance was 14 and the membrane area was 1.54 cm^2^. The change in TEER values for each insert was calculated using the following formula: change in TEER (%) = TEER (Ω cm^2^)/initial TEER (Ω cm^2^) - 100.

### mRNA expression

AGS cells were seeded into six-well plates at 2×10^4^ cells/well in 2 ml medium. Cells were incubated for 24 h and the medium was replaced with SFM. Following 24 h, the medium was replaced again with SFM containing Cf-GP (0, 5, 10 or 20 μg/ml) for 24 h. Cells were treated with the TRIzol reagent (Invitrogen Life Technologies, Carlsbad, CA, USA) and the RNA extracted was quantified using Oligo(dT) primers (Intron Biotechnology Co. Ltd., Seongnam, Korea); the corresponding cDNA was then synthesized. cDNA was subjected to amplification using a PCR kit (dNTP mix, 10X *Ex Taq* Buffer and *Ex Taq*; Takara Bio, Inc., Shiga, Japan) with primers (Table I) in 0.1% diethylpyrocarbonate water. PCR products were resolved on 1% agarose gels. Gels were stained with 10 mg/ml ethidium bromide to visualize amplification products.

### Western blot analysis

AGS cells were cultured in 100-mm diameter dishes. Cells were cultured to 60–80% confluence and the medium was replaced with SFM for 4 h. Next, the medium was replaced with fresh SFM containing Cf-GP (0, 5, 10 or 20 μg/ml) for 24 h. Cells were washed with phosphate-buffered saline and added to lysis buffer [50 mM Tris-HCl (pH 7.4), 150 mM NaCl, 1 mM EGTA, 1% NP-40, 1 mM NaF, 1 mM Na_3_VO_4_, 1 μg/ml aprotinin, 1 μg/ml leupeptin, 1 μg/ml pepstatin A, 0.25% Na-deoxycholate and 1 mM PMSF]. Lysates were separated using 10–15% SDS-PAGE and transferred onto polyvinylidene fluoride membranes (Millipore, Billerica, MA, USA). The membranes were blocked with 1% bovine serum albumin in TBS-T [10 mM Tris-HCl (pH 7.5), 150 mM NaCl and 0.1% Tween 20] at room temperature and incubated with agitation with specific antibodies: anti-claudin-1, −2, −3 and −4 (1:1,000), anti-β-catenin (1:1,000), anti-E-cadherin (1:1,000), anti-MMP-2 and −9 (1:1,000) and anti-tissue inhibitors of metalloproteinases-1 (TIMP-1; 1:1,000; all from Santa Cruz Biotechnology, Inc., Santa Cruz, CA, USA). The secondary peroxidase-conjugated goat, mouse and rabbit antibodies (1:10,000) were purchased from GE Healthcare Bio-Sciences (Piscataway, NJ, USA). Bands were visualized using Super Signal West Pico Stable Peroxide solution and the Super Signal West Pico Luminol/Enhancer solution (Pierce Biotechnology, Inc., Rockford, IL, USA) and developed using Kodak X-ray film (Eastman Kodak Company, Rochester, NY, USA).

### Gelatin zymography

AGS cells were cultured in six-well plates to 60–80% confluence and the medium was replaced with SFM for 4 h. Next, the medium was replaced with fresh SFM containing Cf-GP (0, 5, 10 or 20 μg/ml) for 24 h. The obtained conditioned medium was loaded in 10% SDS-free acrylamide gels with 0.1% gelatin. The completed loading gel was treated with 2.5% Triton X-100 and incubated under agitation for 30 min, followed by incubation with developing buffer (50 mM Tris-HCl, 150 mM NaCl, 5 mM CaCl_2_ and 1 μM ZnCl_2_; pH 7.5) at 37°C for 2 days. The gel was fixed (7% acetic acid) and stained (0.5% Coomassie Brilliant Blue 250 in dilute fixing solution).

### Statistical analysis

Data are presented as the mean ± SD and were calculated using SPSS version 10.0 (SPSS, Inc., Chicago, IL, USA). Data were validated by analysis of variance (ANOVA). P<0.05 was considered to indicate a statistically significant difference and was determined by Duncan's multiple range test for group comparisons.

## Results

### Cf-GP inhibits AGS cell proliferation

**Figure 1:**
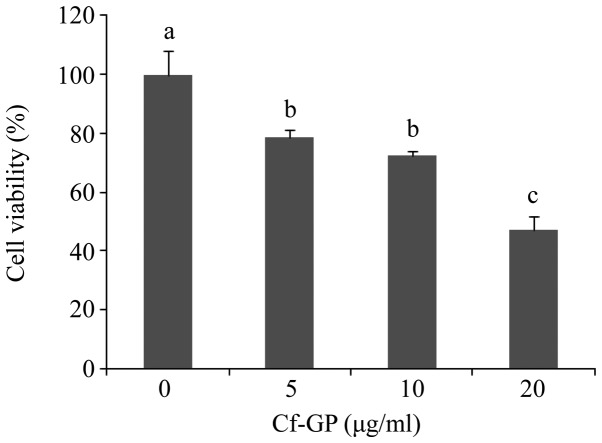
Cf-GP reduces the viability of AGS human gastric cancer cells. AGS cells were treated with 0, 5, 10 or 20 μg/ml Cf-GP for 24 h. Cell viability was measured using the MTS assay. Data are presented as the means ± SD; P<0.05 by ANOVA. Values with different letters are significantly different according to Duncan's multiple range test. Cf-GP, *Capsosiphon fulvescens* glycoprotein. MTS, 3-(4,5-dimethylthiazol-2-yl)-5-(3-carboxymethoxy-phenyl)-2-(4-sulfonyl)-2H-tetrazolium.

MTS assays were used to investigate the effect of Cf-GP (0, 5, 10 or 20 μg/ml) on AGS cell proliferation. Cf-GP at 20 μg/ml resulted in a 50% decrease in proliferation (Fig. 1). In addition, Cf-GP inhibited AGS cell growth in a dose-dependent manner. We previously reported that Cf-GP induced proliferation of human intestinal epithelial IEC-6 cells (17).

### Cf-GP inhibits AGS cell invasion

**Figure 2:**
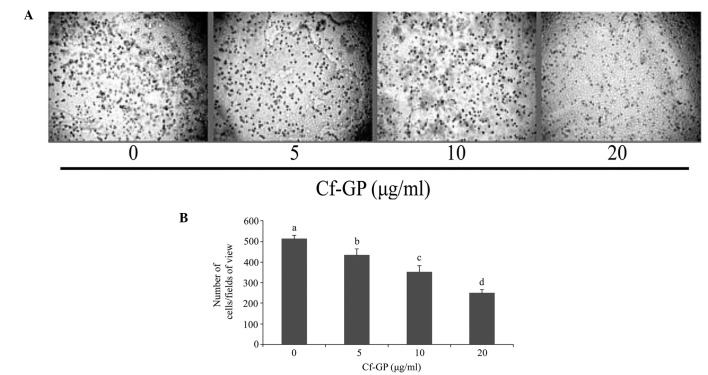
Inhibition of AGS cell invasion by Cf-GP. (A) Cells were seeded into Matrigel chambers for 24 h. The medium was replaced with SFM plus Cf-GP (0, 5, 10 or 20 μg/ml) for 24 h. Invading cells on the bottom filter were stained with hematoxylin. (B) Cells in a random fixed area were counted. Cf-GP, *Capsosiphon fulvescens* glycoprotein; SFM, serum-free media. Data are presented as the means ± SD; P<0.05 by ANOVA. Values with different letters are significantly different according to Duncan's multiple range tests.

In general, increased proliferation of cancer cells to other tissues is a result of accelerated invasion. Since Cf-GP inhibited AGS cell growth, the effect of Cf-GP on invasion was investigated using hematoxylin staining (Fig. 2A). As demonstrated in Fig. 2B, the Cf-GP treatment group exhibited decreased cell invasion compared with control cells. Treatment with 20 μg/ml Cf-GP led to a 50% reduction compared with the control group.

### Cf-GP increases TEER values in AGS cells

**Figure 3:**
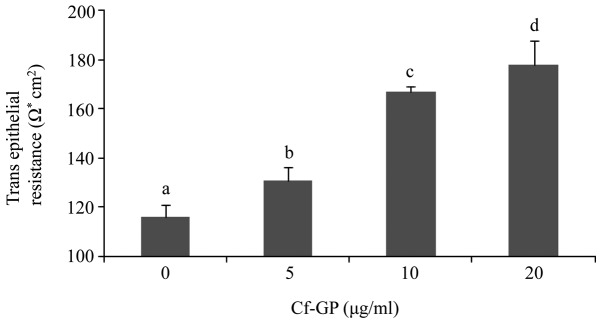
Increased TEER by Cf-GP in AGS cells. Cells were treated with various concentrations of Cf-GP (0, 5, 10 or 20 μg/ml) for 24 h. The lower chamber was maintained with 10% FBS. Following 24 h, the upper chamber was separated and TEER values determined using the voltohmmeter. Data are presented as the mean ± SD; P<0.05 by ANOVA. Values with different letters are significantly different according to Duncan's multiple range tests. TEER, transepithelial electrical resistance; Cf-GP, *Capsosiphon fulvescens* glycoprotein; FBS, fetal bovine serum.

Increased cell membrane permeability is mediated by weakening of TEER. We thus evaluated the effect of Cf-GP on AGS cell invasion by measuring TEER. Decreases of TEER values allowed easier penetration of intracellular material. Normal cells indicate higher TEER values by the strong electrical resistance of the cell membrane. However, MMP activity induced degradation of cell membranes and permeability in cancer cells. Therefore, cancer cells have lower TEER values and this increases invasion. In the present study, TEER values in AGS cells were increased upon Cf-GP treatment in a dose-dependent manner (Fig. 3).

### Effect of Cf-GP on TJ- and metastasis-associated protein expression

**Figure 4:**
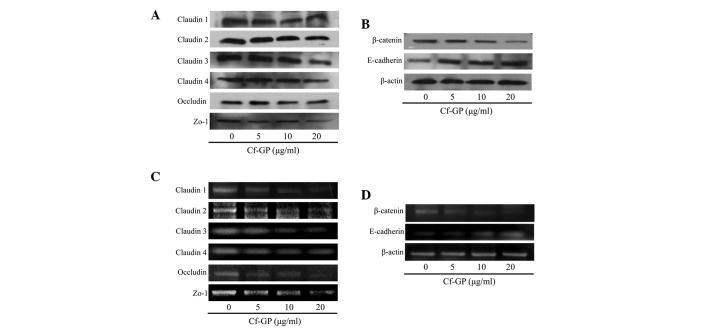
Effect of Cf-GP on TJ proteins in AGS cells. Protein expression was examined by western blot analysis and cDNAs were subjected to PCR. Reaction products were electrophoresed in a 1% agarose gel and visualized by ethidium bromide staining. (A) Expression of TJ proteins were decreased upon incubation with Cf-GP for 24 h. (B) mRNA levels were also decreased, as revealed by RT-PCR. (C) Decreased levels of cell adhesion protein (β-catenin) and increased E-cadherin, which inhibits β-catenin. (D) Consistent β-catenin and E-cadherin results at the mRNA level as determined by RT-PCR. Cf-GP, *Capsosiphon fulvescens* glycoprotein; TJ, tight junction.

The increased membrane permeability in cancer cells is due to mutations in a variety of genes associated with TJ proteins. Typically, overexpression of TJ proteins (claudin, zo-1 and occludin) and TJ-related proteins (β-catenin) is observed in cancer cells. In addition, β-catenin levels are increased due to loss of E-cadherin. Therefore, β-catenin increases cancer cell adhesion. In the present study, protein and mRNA levels were determined by western blot analysis and RT-PCR, respectively. As demonstrated in Fig. 4A and B, in control cells, expression levels of the TJ proteins, claudin, zo-1 and occludin increased, but were reduced in a dose-dependent manner upon Cf-GP treatment. In addition, β-catenin levels were reduced while that of the inhibitor of β-catenin, E-cadherin, was increased by Cf-GP treatment at the protein and mRNA levels (Fig. 4C and D).

### Cf-GP inhibits MMP activity and expression

**Figure 5:**
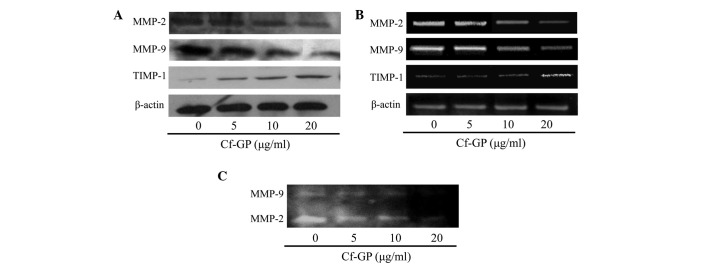
Effect of Cf-GP on MMP and TIMP-1 protein expression in AGS cells. (A and B) Cells were treated with 0, 5, 10 or 20 μg/ml Cf-GP for 24 h. Western blot analysis and RT-PCR were performed as described. (C) AGS cells were cultured in six-well plates and treated with Cf-GP (0, 5, 10 or 20 μg/ml) for 24 h and analyzed by gelatin zymography. Cf-GP, *Capsosiphon fulvescens* glycoprotein; MMP, matrix metalloproteinase; TIMP, tissue inhibitors of metalloproteinases.

The interaction between cancer cells and the basement membrane is a major step in metastasis and invasion (18). The basement membrane is composed of substances, including collagen and lamin. Cell membrane degradation by MMP proteins and cancer cells is an important process for invasion and metastasis. In particular, MMP-2 and MMP-9 are important mediators of basement membrane degradation of gelatin and factors involved in angiogenesis and cancer cell invasion are known to induce MMP-2 and −9 (19). Thus, inhibition of MMP-2 and −9 is essential for preventing basement membrane destruction. MMP proteins were associated with TJ proteins and upregulation of TJ proteins induced MMP activation in cancer cells. MMP activity leads to cell invasion and metastasis. In general, normal cells have tissue inhibitors of metalloproteinases (TIMPs). Therefore MMP activation and degradation of cell basement membrane are inhibited. However, cancer cells have constant proliferation and invasion by upregulation of TJ proteins and do not have TIMPs (20). In our previous study, expression of TJ proteins was confirmed. Therefore, MMP and TIMP levels were evaluated in AGS cells following treatment with Cf-GP. MMP-2 and −9 levels were decreased due to increased TIMP-1 as determined by western blot analysis and RT-PCR (Fig. 5A and B). In addition, gelatin zymography assays indicated that MMP protein levels decreased with increasing Cf-GP concentrations (Fig. 5C).

## Discussion

Vividiffusion, division and gene expression are maintained by homeostasis in normal cells. However, in cancer cells, homeostasis is not maintained, enabling mutations to develop and invasion to continue. Therefore, cancer cell metastasis and invasion must be inhibited. MMP and TJ proteins are important for cancer cell invasion and metastasis, and are closely associated with cell membrane permeability (21). Invasion and metastasis of cancer cells occurs through degradation of the cell basement membrane by upregulation of MMP proteins (22). Cancer cell adhesion is affected by overexpression of proteins associated with cell membrane permeability. Increased MMP expression correlates with cancer cell metastasis and invasion (23,24). In addition, expression of adhesion proteins is important for loss of invasion and metastatic capacities. TJ proteins are key for the passage through epithelial and endothelial barriers as well as in osmoregulation and maintenance of cell polarity (25–27). Therefore, cancer cell membrane permeability is weakened due to overexpression of TJ and MMP proteins, resulting in enhanced metastasis and invasion. Expression levels of the TJ proteins, claudin, zo-1 and occludin, are increased, resulting in the inhibition of invasion and metastasis by cancer cells (28,29). Changes in cell membrane permeability are induced by increased expression of claudin, zo-1 and occludin. In addition, TEER values are decreased by overexpression of TJ proteins (30). Therefore, suppression of claudin, zo-1 and occludin is important for inhibition of metastasis and invasion. In addition, increased expression of β-catenin facilitates invasion and metastasis of cancer cells, which is mediated by the simultaneous actions of MMP and TJ proteins. Thus, expression of β-catenin and E-cadherin is important (31,32). In normal cells, E-cadherin inhibits β-catenin, which is associated with cell adhesion, inhibiting its nuclear accumulation. Since E-cadherin expression is decreased in cancer cells, β-catenin accumulates in the nucleus, resulting in enhanced adhesion. Cell membrane permeability is impeded by increased MMP and TJ expression, while adhesion is enhanced, resulting in the rapid proliferation of cancer cells. Previous studies using Cf-GP revealed that apoptosis of AGS cells was associated with Fas signaling (17) and that β-catenin expression was reduced during apoptosis. Therefore, the effect of Cf-GP on AGS human gastric cancer cell invasion and metastasis was investigated.

Cf-GP treatment (0, 5, 10 or 20 μg/ml) for 24 h inhibited the growth of AGS cells. The Cf-GP-treated group exhibited decreased cell invasion and increased TEER. The latter is based on the electrical resistance of the cell membrane and is closely associated with cell invasion due to its association with cell membrane permeability. Next, the expression levels of associated proteins, MMPs, TIMP-1, claudin, zo-1, occludin, β-catenin and E-cadherin, were evaluated. Dose-dependent decreases in MMP expression and increases in TIMP-1 expression, an MMP inhibitor, were identified following Cf-GP treatment for 24 h at the protein and mRNA levels. In addition, MMP-2 and −9 levels were observed by gelatin zymography, confirming inhibition at the protein level. As a result, compared with the control group, Cf-GF-treated cells exhibited significantly decreased claudin, zo-1 and occludin protein and mRNA levels. This result confirmed that suppression of cancer cell invasion is mediated by inhibition of TJ protein expression.

In summary, Cf-GP decreased MMP expression in a dose-dependent manner. MMP expression is associated with cell basement membrane degradation factor and inhibition of the TJ proteins involved in membrane permeability. Inhibition of AGS growth and invasion was confirmed. Anti-tumor activity in various cell lines using marine algae has been previously reported and results of the present study indicate the promise of functional therapeutics using Cf-GP.

## Figures and Tables

**Table I:** Oligonucleotide sequences of the primer pairs used for RT-PCR.

	Sequence of primers (5′-3′)	
	
Name	Sense	Antisense
MMP-2	GGC-CCT-GTC-ACT-CCT-GAG-AT	GGC-ATC-CAG-GTT-ATC-GGG-GA
MMP-9	CGG-AGC-ACG-GAG-ACG-GGT-AT	TGA-AGG-GGA-AGA-CGC-ACA-GC
TIMP-1	TGG-GGA-CAC-CAG-AAG-TCA-AC	TTT-TCA-GAG-CCT-TGG-AGG-AG
β-catenin	GAA-ACG-GCT-TTC-AGT-TGA-GC	CTG-GCC-ATA-TCC-ACC-AGA-GT
E-cadherin	GAA-CAG-CAC-GTA-CAC-AGC-CCT	GCA-GAA-GTG-TCC-CTG-TTC-CAG
Claudin-1	TCA-GCA-CTG-CCC-TGC-CCC-AGT	TGG-TGT-TGG-GTA-AGA-GGT-TGT
Claudin-2	ACA-CAC-AGC-ACA-GGC-ATC-AC	TCT-CCA-ATC-TCA-AAT-TTC-ATG-C
Claudin-3	AAG-GCC-AAG-ATC-ACC-ATC-GTG	AGA-CGT-AGT-CCT-TGC-GGT-CGT
Claudin-4	TGG-ATG-AAC-TGC-GTG-GTG-CAG	GCA-GAA-GTG-TCC-CTG-TTC-CAG
Occludin	TCA-GGG-AAT-ATC-CAC-CTA-TCA-CTT-CAG	CAT-CAG-CAG-CAG-CCA-TGT-ACT-CTT-CAC
Zo-1	GAA-GCT-TCA-TCT-CCA-GTC-CCT	TGG-GTA-GGG-CTG-TTT-GTC-ATC-ATA
β-actin	CGT-ACC-ACT-GGC-ATC-GTG	GTG-TTG-GCG-TAC-AGG-TCT-TTG

MMP, matrix metalloproteinase; TIMP, tissue inhibitors of metalloproteinases.
